# Supplementary material for: In-vivo measurement of radio frequency electric fields in mice brain
Source: Biosens Bioelectron X. Author manuscript; Available in PMC 2023 Sep 1. (PMC10465067; doi:10.1016/j.biosx.2023.100328)
Supplement: In-vivo measurement of radio frequency electric fields in mice brain [file NIHMS1899098-supplement-In-vivo_measurement_of_radio_frequency_electric_fields_in_mice_brain.pdf]

SUPPLEMENTARY INFORMATION

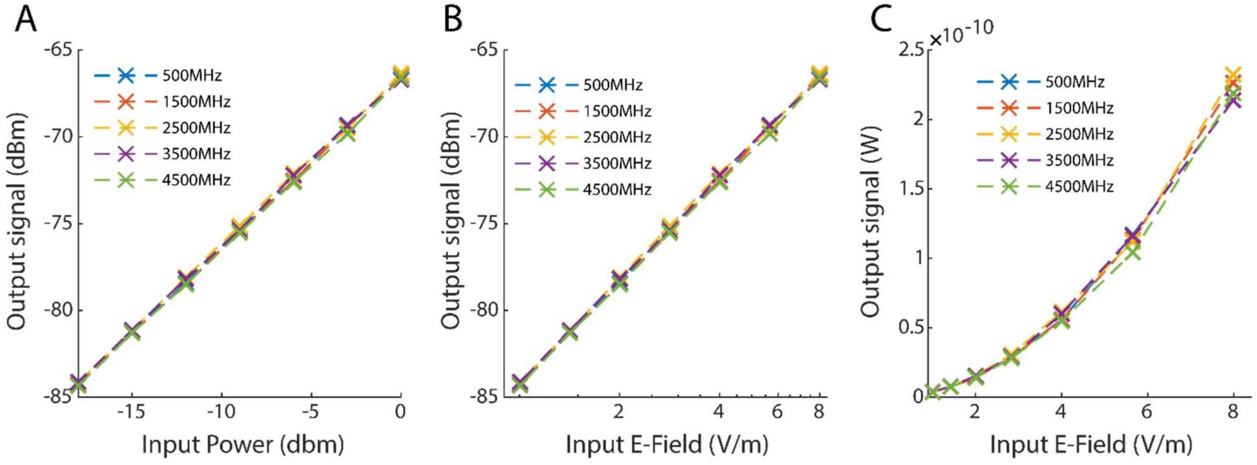

Supplementary Figure 1. **E-field measurement system (input-power, output-power) linearity test at different frequencies. A)** Output signal power in dbm as a function of input power to the antenna in dbm. **B)** Output signal power in dbm as a function of input E-field (V/m) induced by the input power to the antenna (as in A). Note that the x-axis is in logarithmic scale. **C)** Same as B except that the output signal power is plotted in Watts. Note that 1) all three plots show the same data; 2) the measurements are performed with a E-field probe in the center of one chamber of a TEM cell with 28 mm septum height in air; 3) the output power reflects non-calibrated measured values at the spectrum analyzer, the input power is the applied power to the input of the TEM cell and the E-field is the corresponding E-field value in air at the location of the probe.

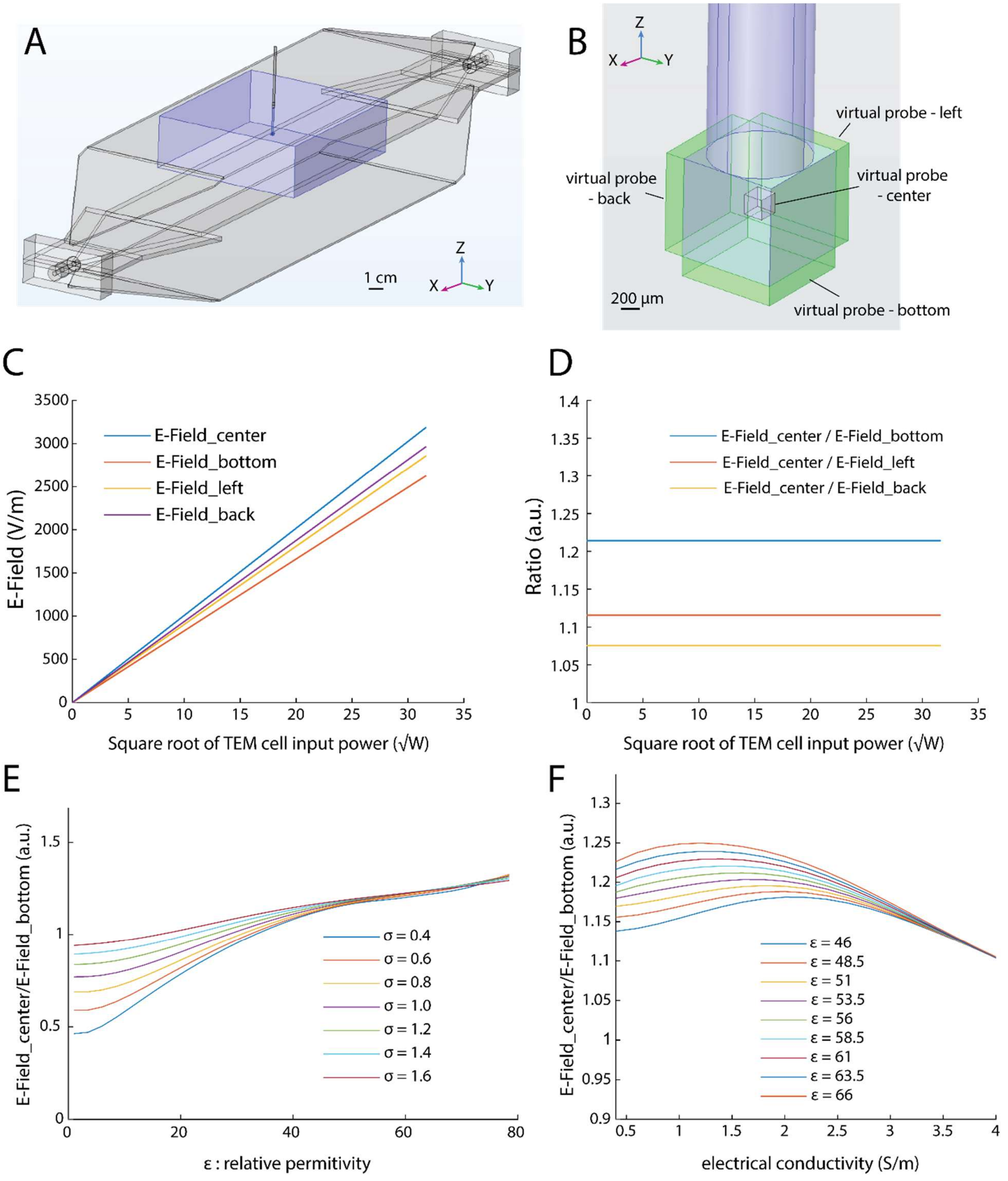

Supplementary Figure 2. **Numerical simulations. A)** 3D drawing of the simulation set-up mimicking the calibration process for the normal probe including a TEM cell with the reservoir of the medium of interest (in blue). **B)** Zoomed-in view of the inserted probe (as in A) with the defined E-Field measurement probing volumes at the center and adjacent to the BSO crystal. Note that here 'virtual probe' regions (green) represent simulation tools to measure the average value of a parameter (here the E-field) across a defined volume. **C and D)** Measured electric-

filed values for all probing volumes show a linear relationship with the square root of the input power to the TEM cell (using the electrical conductivity of 1.06 (S/m) and relative permittivity of 57.77). **E)** Variation of the ratio between the E-field at the center of the BSO crystal and the in the probing volume beneath it (this is the area that the normal probe is supposed to monitor) as a function of the relative permittivity of the medium of interest. Note the relatively low variability of this ratio for different electrical conductivity of the medium at relative permittivity values around that of the brain tissue (~57). **F)** Same measure as in E but as a function of the electrical conductivity of the medium of interest. Note the relatively low variability of this ratio for different relative permittivity of the medium at electrical conductivity values around that of the brain tissue (1 S/m).

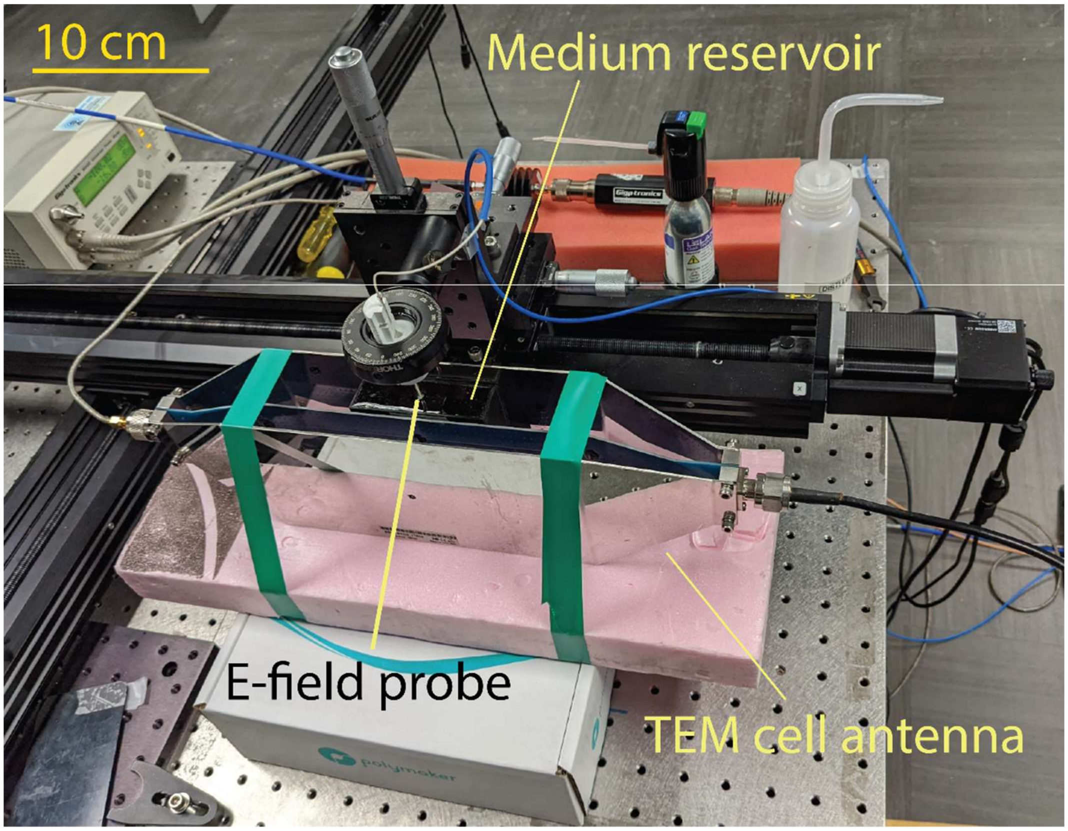

Supplementary Figure 3. **Probe calibration setup.** Photo showing the calibration set-up configuration for the tangential probe using a TEM cell antenna. Here, the medium reservoir is filled with phantom material preparation.

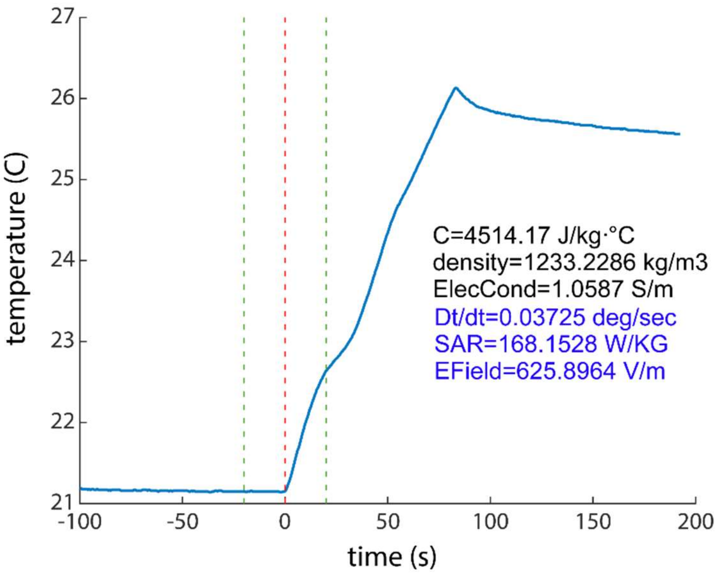

Supplementary Figure 4. **Temperature measurement for probe calibration in Phantom preparation.** Example temperature measurement for E-Field estimate calculation for calibration of E-Field probes immersed in a reservoir filled with a brain phantom preparation in a TEM cell.

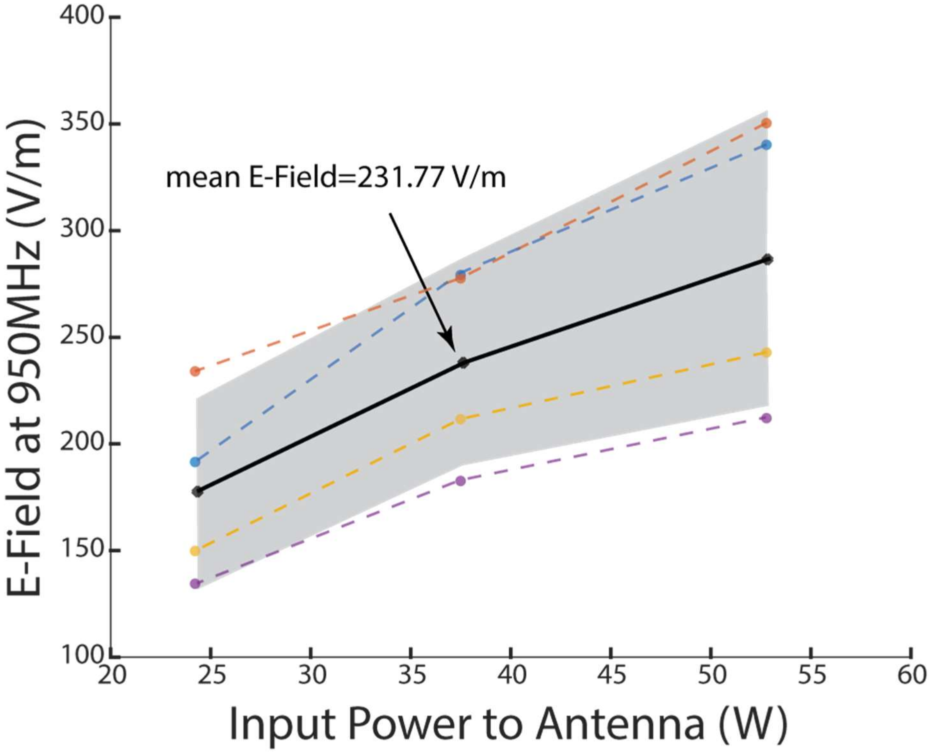

Supplementary Figure 5. **E-field estimate from in-vivo temperature measurements.** Data from (Yaghmazadeh2022). Different color lines denote data from different mice. Solid black line shows mean and gray shade show +/-1 standard deviation from the mean.
